# Aesthetic Reconstruction of Fingertip Defect Using Second Toe Pulp Free Flap

**DOI:** 10.3390/jcm14165855

**Published:** 2025-08-19

**Authors:** Soyeon Jung, Sodam Yi, Seungjun Lee, Seokchan Eun

**Affiliations:** 1 Department of Plastic and Reconstructive Surgery, Hallym University College of Medicine, Hallym University Dongtan Sacred Heart Hospital, Hwaseong 18450, Republic of Korea; ps.soyeon.jung@gmail.com; 2 Department of Plastic and Reconstructive Surgery, Seoul National University College of Medicine, Seoul National University Bundang Hospital, Seongnam 13620, Republic of Korea; lsds4774@snubh.org; 3 Department of Plastic and Reconstructive Surgery, Armed Forces Capital Hospital, Seongnam 13620, Republic of Korea; winters88@naver.com

**Keywords:** fingertip defect, free toe pulp flap, microvascular reconstruction

## Abstract

**Background:** Varioaus methods are available to address fingertip injuries, which are becoming increasingly common. Coverage should ideally involve both functional and aesthetic improvements. The second toe pulp-free flap is useful because of its similarity to the fingertips in shape, texture, and sensation. Herein, we present our clinical experience and surgical methods for fingertip defect reconstruction using second toe pulp-free flaps. **Materials and Methods:** Between April 2022 and May 2023, 13 toe pulp-free flaps were used to reconstruct fingertip defects. The average patient age was 50.1 years (range, 35–67 years), and nine of the 13 patients were male. Nine patients were injured on the right hand, and four on the left hand. After complete debridement, a toe-pulp flap was harvested with a teardrop from the ipsilateral side. The cases included the reconstruction of four index fingers, seven middle fingers, and two little fingers. Functional and aesthetic assessments were performed postoperatively. **Results:** All flaps survived completely, with no partial necrosis. The average flap size was 1.5 × 2 cm (range, 0.8 × 1.5 to 2.0 × 3.0 cm). None of the patients had functional impairments. No emergency surgeries were required during the follow-up period. The median follow-up period was 28 months, and the median duration of surgery was 119 min (range, 100–140 min). The average static two-point discrimination score for the injured finger pulp was 3.7 mm (range, 2–5 mm), the Quick Dash score was 3.4 (range, 2.3–4.2), and the Vancouver scar scale was 1.5 (range, 0–2). **Conclusions:** The toe pulp-free flap is the optimal choice for surgical treatment of fingertip defects and injuries, with excellent functional and cosmetic results.

## 1. Introduction

The importance of functional and aesthetic hands cannot be overstated. Among traumatic hand injuries, fingertip injury with a pulp defect is very common. When left untreated, its prognosis is unsatisfactory [1]. Many treatment options for fingertip defects exist, including primary closure, skin grafting, and local, distant, and free flap transfer. However, the optimal treatment for this injury remains controversial.

Reconstruction of fingertip defects requires improvements in functional outcomes and an acceptable appearance of the hand. The choice of reconstructive options can vary depending on the need to resurface the finger and size of the defect, ranging from simple skin grafting and local flaps to complex microsurgical procedures [2,3]. Flap transfer to the fingertip has shown remarkable outcomes with optimal morbidity. When considering the theory of “like with like,” the transferred tissue must provide similar texture, tenacity, and thin subcutaneous fat of the pulp [4,5]. Therefore, the toe pulp is strongly suggested for resurfacing the fingertip pulp defect [2,3,4].

The main theme of this study was to present cases of toe pulp-free transfer for reconstructing fingertip defects in an aesthetically pleasing manner. The sensitivity, appearance, and function of the injured fingers were assessed during follow-up examinations.

## 2. Materials and Methods

Between January 2022 and June 2023, we performed 13 fingertip reconstructions using second toe pulp-free flaps. A chart review, including demographic and medical data, was conducted. Thirteen patients (nine men and four women) with an average age of 50.1 years (range, 35–67 years) were enrolled. The modes of injury included crushing, cutting, and infection. The timing of reconstruction was dependent on the wound status assessment. Some patients immediately underwent reconstructive surgery, whereas others underwent delayed surgery. Among the patients who received second toe pulp free flap transfer to the finger, those suffering from the apical fingertip defect due to injury were included. Any other type of defect or flap transfer was excluded. Seven patients were affected on the right hand and six on the left. The number of affected fingers was four for the second finger, seven for the third finger, and two for the fifth finger. After removing the injured tissue, a teardrop-shaped flap was harvested from the ipsilateral second toe. The mean size of the finger pulp defects after debridement was 2.8 × 1.6 cm (range, 1.4 × 1.8 to 3.2 × 2.5 cm). The donor site was closed primarily in all patients. Post-operative evaluations were conducted to elucidate functional and aesthetic improvements (Table 1).

### Surgical Methods

Surgery was performed under general anesthesia and aided by the application of a pneumatic tourniquet. The dissection and microsurgical procedure required loupe magnification and a surgical microscope. The procedure started with adequate debridement under a finger tourniquet, and subsequently, the recipient vessels of the digital artery and volar vein were prepared. When dissecting the digital veins, the volar side of the finger was initially explored. If an appropriate vein could not be found on the volar side, it was prepared on the dorsal side. After completion of the recipient vessel and nerve preparation, the finger tourniquet was removed. Skin marking for the toe-pulp flap was continued on the second toe according to the size and shape of the fingertip defect.

Dissection of the vein was initiated by paying attention to obtaining sufficient length. This procedure should be performed under magnification to minimize venous damage. The dissection went deep into the extensor tendon layer and was elevated from the distal to the proximal direction by releasing the septa. Dissection was continued proximally, while preserving the neurovascular bundles.

After the toe-pulp flap was completely raised, the donor site was closed. The raised toe pulp tissue was transferred to the recipient site. Microanastomosis was performed in an end-to-end fashion in both arteries and veins. The size of the digital artery and volar veins was 1–1.5 and 0.6–1.2 mm, respectively. An arterial anastomosis was created between the proper digital artery of the injured finger and the plantar digital artery of the flap. Venous anastomosis was performed between the local veins around the defect of the injured finger and the dorsal metatarsal veins of the flap. The appropriate digital nerves of the flap were anastomosed to those of the injured finger. Microanastomoses of the vessels and nerves were performed using 10–0 nylon sutures. Neurorrhaphy was performed in an end-to-side manner, so as not to injure the original recipient nerve fiber. It was as close to the flap as possible so that the nerve regenerated quickly. Vein insufficiency frequently occurs when the remaining soft tissue in the teardrop-shaped proximal area of the flap is pressurized. The use of skin grafting rather than suturing under tension is recommended to avoid this. Postoperative flap monitoring included capillary refill, color, temperature, and turgor. Motion and exercise may be gradually started when the flap stabilizes 2 weeks after surgery.

All patients were assessed at 12 months using the Quick Disabilities of the Arm, Shoulder, and Hand (Q-DASH) scores. In addition, the static two-point discrimination (2PD) and Semmes–Weinstein monofilament tests were applied to measure the sensitivity of the recipient sites. A visual analog scale ranging from 0 to 10 was used to estimate the degree of pain in the affected finger and donor site. Assessment for subjective aesthetic appearance was also conducted.

## 3. Results

All flaps survived completely, with no partial necrosis. The average flap size was 1.5 × 2 cm (range, 0.8 × 1.5 to 2.0 × 3.0 cm). None of the patients showed functional impairments. No intervention or revision surgery was required during the perioperative period. The median follow-up period was 28 months, and the median duration of surgery was 119 min (range, 100–140 min). The average static two-point discrimination score for the injured finger pulp was 3.7 mm (range, 2–5 mm), the Quick Dash score was 3.4 (range, 2.3–4.2), and the Vancouver scar scale was 1.5 (range, 0–13). Problems with gait disturbances and complaints of joint stiffness at the donor site were not addressed (Table 1).

### 3.1. Case Description

#### 3.1.1. Case 1

**Figure 1:**
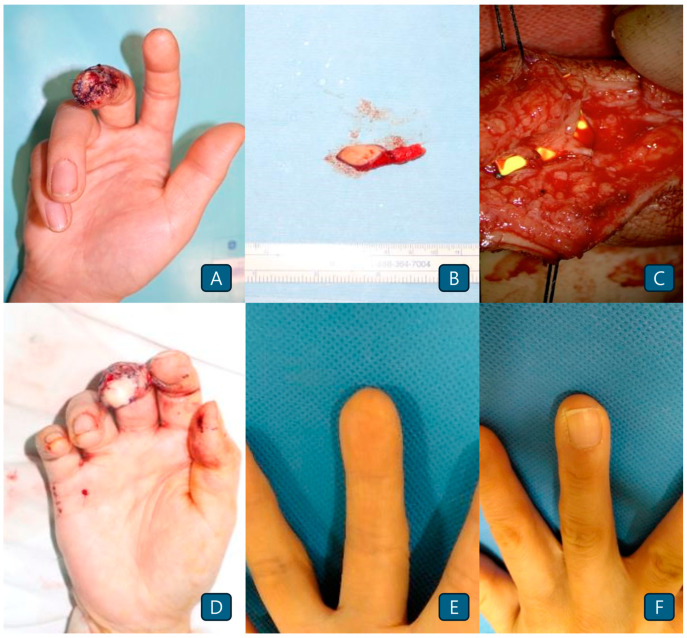
(**A**) The apical tip of the right third distal phalanx underwent crushing injury with no nail bed defects. (**B**) Toe pulp-free flap elevated from the ipsilateral second toe. (**C**) The digital arteries of the left second finger and volar vein were sutured with a second toe pulp flap pedicle using an end-to-end anastomosis. Nerve anastomosis was done in an end-to-side manner. (**D**) Immediate postoperative image. (**E**) Postoperative photograph obtained 5 years after surgery on the volar side. (**F**) Postoperative photograph obtained 5 years after surgery on the dorsal side.

A 41-year-old female patient presented with an occupational injury to the right third finger and loss of the apical pulp (Figure 1A). A toe pulp flap measuring approximately 1.5 × 2 cm was elevated from the ipsilateral second toe (Figure 1B). Arterial and venous anastomoses were performed on the second toe-pulp flap pedicle in an end-to-end manner (Figure 1C). Otherwise, the nerve anastomosis was done in an end-to-side manner. The operative time was approximately 125 min. The patient recovered with no functional or cosmetic complication until the time of 60-month postoperative observation (Figure 1D–F).

#### 3.1.2. Case 2

**Figure 2:**
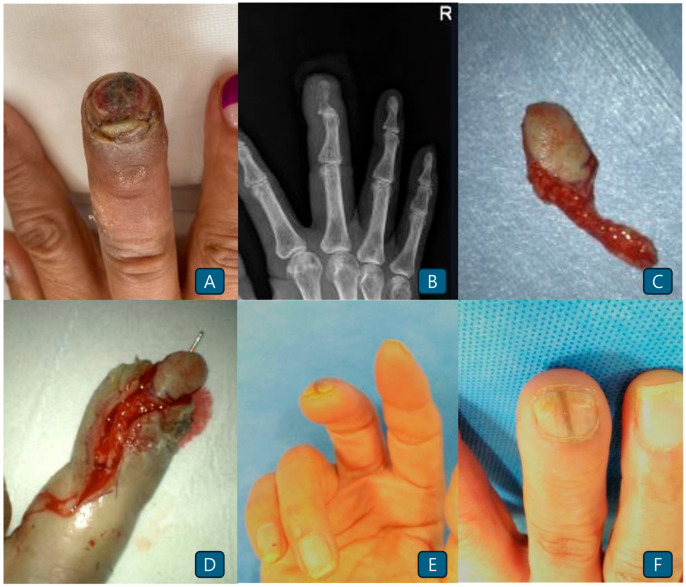
(**A**) The tip of the right third distal phalanx shows skin loss involving a moderately sized nail-bed defect. (**B**) Distal phalangeal fractures caused by the same injury. (**C**) Elevated toe pulp flap from the second toe. (**D**) The pedicle is connected to the recipient’s digital artery. (**E**) Postoperative photograph obtained 1 year after surgery (apical view). (**F**) Postoperative photograph obtained 1 year after surgery (dorsal view).

A 67-year-old female patient visited the clinic with a right third fingertip fracture and a pulp defect caused by machinery injury (Figure 2A,B). The fingertip injury included a pulpal defect with a moderate-sized nail bed injury. A toe pulp flap was elevated from the second toe to the same site (Figure 2C). Flap transfer and microanastomosis of the vessels and nerves were successfully performed (Figure 2D). The operative time was approximately 140 min. The postoperative results showed a well-settled flap with good sensation and aesthetics (Figure 2E,F).

#### 3.1.3. Case 3

**Figure 3:**
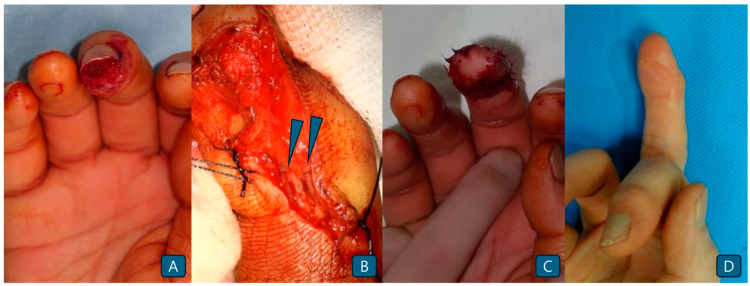
(**A**) The injured right third fingertip showing a skin defect invading the pulp. (**B**) Anastomosis between the flap pedicle and digital vessels. (blue arrow heads). (**C**) Immediate postoperative photograph. (**D**) Postoperative photograph of the volar side 8 months after surgery.

A 54-year-old man sustained an injury to the right middle finger while working in a factory (Figure 3A). The tip of the distal phalanx showed a 1.5 × 2 cm-sized skin defect with no nail bed injury. Toe-pulp flap elevation and microanastomosis were successfully performed (Figure 3B). While suturing the flap, the area of the pedicle was covered with a skin graft harvested from the hypothenar area to release tension (Figure 3C). Functional and cosmetic outcomes were satisfactory at the 8-month postoperative follow-up. No limitations in finger flexion or extension were observed (Figure 3D).

#### 3.1.4. Case 4

**Figure 4:**
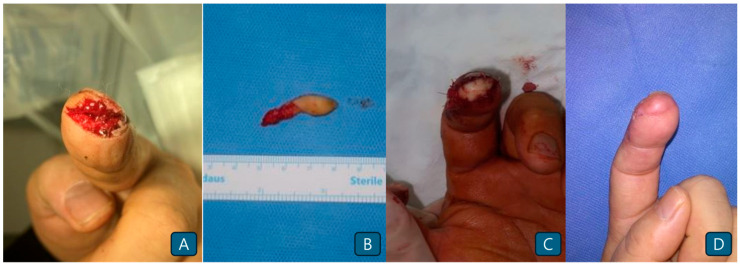
(**A**) Fingertip injury with apical defect. (**B**) A small toe pulp flap is elevated from the ipsilateral second toe. (**C**) Immediate postoperative photographs. (**D**) Postoperative photograph obtained 3 months after surgery.

A 62-year-old female patient sustained a glass injury to her left index fingertip. Loss of the skin envelope with no nail bed defects was observed on the fingertips (Figure 4A). A 0.8 × 1.5-cm-sized toe pulp flap from the ipsilateral second toe was transferred to the defect (Figure 4B). Microanastomosis of the artery and neurorrhaphy were performed without venous anastomosis because no suitable veins were identified. The flap survived without any events, and the final outcome was satisfactory (Figure 4C,D).

**Table 1:** Patient demographics and functional outcomes.

Patient No	Sex	Age	Location (Finger)	Days After Trauma	Flap Size (cm × cm)	Duration of Surgery (min)	Anastomosis (A; Artery, V; Vein, N; Nerve)	Skin Graft (Yes or No)	Follow-Up (Months)	2-Point D	Q-DASH	Vancouver Scar Scale
1	M	58	Lt 3rd	4	1.5 × 2	120	1 A, 1 V, 1 N	N	45	4	4	1
2	F	41	Rt 3rd	3	1.5 × 2	125	1 A, 1 V, 1 N	N	60	2	3	0
3	M	46	Rt 5th	6	2 × 3	120	1 A, 1 V, 1 N	Y	35	4	4.2	2
4	M	45	Rt 2nd	3	1.5 × 2	110	1 A, 1 V, 1 N	N	30	4	3.2	4
5	F	67	Rt 3rd	12	1.5 × 2	140	1 A, 1 V, 1 N	N	12	4	3	0
6	M	45	Rt 3rd	3	2 × 3	120	1 A, 1 N	Y	25	5	4	2
7	M	52	Lt 2nd	4	1.5 × 2	110	1 A, 1 V, 1 N	N	22	4	3.4	1
8	M	57	Rt 3rd	3	2 × 3	120	1 A, 1 V, 1 N	N	14	4	4	2
9	M	54	Rt 3rd	6	1.5 × 2	110	1 A, 1 V, 1 N	N	8	3	2.4	0
10	M	45	Rt 2nd	3	1.5 × 2	120	1 A, 1 V, 1 N	N	15	4	4	2
11	F	62	Lt 2nd	2	0.8 × 1.5	130	1 A, 1 N	N	3	2	2.3	1
12	M	45	Rt 3rd	5	1.5 × 2	125	1 A, 1 V, 1 N	Y	16	4	3	2
13	F	35	Lt 5th	3	1.5 × 2	100	1 A, 1 V, 1 N	N	24	4	4	2

## 4. Discussion

Reconstruction of the fingertip pulp remains challenging for surgeons because of the paucity of adjacent tissue, wherein the goal should include both restoration of function and aesthetics. The hands are one of the most important parts of the body, both functionally and aesthetically. As such, addressing both of these aspects is critical in reconstruction post-injury. Several surgical methods are available today for surgeons to accomplish both acceptable functionality and favorable appearance of the hand in their patients [2,3,6].

Surgical options for fingertip reconstruction vary from local flaps to free flap transfers to restore pulp and volar defects. Local flaps are ideal candidates for functionally adjacent tissues. For instance, the thenar flap or cross-finger flap is a reliable option to restore the fingertip. Easy access and shortened operation time are benefits of the local flap transfer. However, the unlikeness of the tissue, joint stiffness, and the need for a secondary procedure may be drawbacks of the operations [7,8,9]. The advancement flap based on the homodigital artery can restore an acceptable fingertip with local pulp tissue. Unlike the retrograde type of homodigital artery flap, the antegrade type is able to minimize the sacrifice of the digital artery. Nevertheless, the flap needs to be moved from the same finger territory; thus, the size of the flap is limited [10,11]. Especially when treating the sizable defect of the fingertip pulp, the flap should be harvested and mobilized from the more proximal area, resulting in unfavorable reconstruction with unlikeness. Moreover, it needs the pedicle dissection to mobilize the flap, causing contracture and cold intolerance [10,11]. Therefore, the availability of local soft tissues to cover complex fingertip defects is limited [12,13]. Surgeons must also consider local tissues based on the features of the fingertip wound, the patient’s requirements, and the surgeon’s preference for reconstructing the fingertip to minimize deformity and enhance function [14,15]. Considering the primary benefit of the free flap, the donor tissue can be chosen with more freedom. However, drawbacks such as the complexity of the surgical procedure, prolonged operation time, and the risk of vascular crisis and failure exist. Despite these challenges, the experience in handling vessels has increased, facilitating improved outcomes with the development of microsurgery.

Diverse free tissue options exist, which are essential when considering the anatomical restoration of the fingertip with tissue similarity. In addition, the size, location, pliability, and sensation of the transferred tissue should be considered [7,16]. A free thenar flap based on the superficial radial artery has the advantage of easy access during emergency surgery, a texture similar to that of the fingertip, and durable and glabrous skin. However, obtaining a sensory flap is hindered by inconsistent nerve innervation and the relative difficulty of harvesting a small area [17,18,19]. The hypothenar flap also has the advantages of being located in the same operative field, similar to the fingertip tissue, and primary closure of the donor site, because extensive tissue in the hypothenar area can be preserved. However, these flaps exhibit anatomical variations, making it difficult to identify the perforator and cutaneous nerves in the distal ulnar area. Additionally, they are best suited for elliptical finger volar defects, not for fingertip apical defects [20,21]. Considering its anatomical and textural similarities with the fingertip pulp, the toe pulp seems to be the optimal choice for defect reconstruction. The toe pulp can provide sufficient amounts of tissue with an elliptical shape, ideal for fingertip reconstruction. Furthermore, its similarity with fingertip tissue allows for “like with like” reconstruction [22,23]. As a result, color and texture matching can be achieved with anatomical similarity. In terms of contour and thickness, toe pulp tissue is also excellent in terms of aesthetics and function. Moreover, reconstruction of the fingertip using a toe pulp-free flap can help restore sensation [22,23]. Nerve reinnervation is so rapid that sensory recovery is promising [24]. Primary closure of the donor site is also feasible since it is a partial flap that uses only half of the pulp of the second toe. This single-step procedure results in favorable sensory recovery, excellent aesthetic appearance, fingerprint restoration, and satisfactory finger function [4,23].

Despite its advantages, several challenges exist in toe pulp transfer, including the risk of insufficient vessel length, vessel spasms, and tension for closure. The surgical procedure requires precise handling for exploration and preservation of the subcutaneous vein, demanding high levels of surgical experience and skill [25,26]. In the present cases, when a suitable vein could not be found, only an arterial anastomosis was performed. Fortunately, the flaps survived without compromise. To the best of our knowledge, this flap is relatively small, allowing for better imbibition and early marginal vessel ingrowth. Furthermore, the toe pulp is a dense tissue that is resilient to venous congestion [26]. The pedicle sometimes can be exposed or compressed because of the bulkiness of the toe-pulp flap and the limited space of the fingertip. To handle this, the toe-pulp flap needs to be raised as thin as possible, and employing skin grafting on the exposed pedicle is useful to minimize tension. For this, skin can be easily accessed from the hypothenar area [4,27]. Taken together, despite these challenges, the outcomes were excellent, thus highlighting the importance of performing skin grafts whenever necessary.

This study has a limitation due of the small number of cases. However, we conducted postoperative assessments to elucidate the restoration of the function. Another limitation would be the absence of statistical evaluation. The comparison between toe-pulp transfer and others helps verify the priority of choice. We agree with the need for further study with the statistical evaluation.

## 5. Conclusions

Fingertip injuries and defects require reconstructive procedures to restore function and aesthetics. A range of approaches exists because of the unique anatomical and tissue features of fingertips. However, toe pulp best satisfies the requirements for ideal reconstruction. Despite various potential surgical challenges, including vascular crisis and failure, as well as patient outcomes, including motion, appearance, and sensation restoration, are reliable and satisfactory at the appropriate levels of surgical experience and skill.

## Data Availability

Data are contained within the article.

## Abbreviations

The following abbreviations are used in this manuscript:

Q-DASH	Quick Disabilities of the Arm, Shoulder, and Hand scores
2PD	Static two-point discrimination
